# Recurrent probabilistic neural network-based short-term prediction for acute hypotension and ventricular fibrillation

**DOI:** 10.1038/s41598-020-68627-6

**Published:** 2020-07-20

**Authors:** Toshio Tsuji, Tomonori Nobukawa, Akihisa Mito, Harutoyo Hirano, Zu Soh, Ryota Inokuchi, Etsunori Fujita, Yumi Ogura, Shigehiko Kaneko, Ryuji Nakamura, Noboru Saeki, Masashi Kawamoto, Masao Yoshizumi

**Affiliations:** 1 Graduate School of Advanced Science and Engineering, Hiroshima University, 1-4-1 Kagamiyama, Higashi-Hiroshima, Hiroshima, 739-8527 Japan; 2 Graduate School of Engineering, Hiroshima University, 1-4-1 Kagamiyama, Higashi-Hiroshima, Hiroshima, 739-8527 Japan; 3 Academic Institute, College of Engineering, Shizuoka University, 3-5-1, Johoku, Naka-ku, Hamamatsu, Shizuoka, 432-8561 Japan; 4 Department of Emergency and Critical Care Medicine, JR General Hospital, 2-1-3 Yoyogi, Shibuya-ku, Tokyo, 151-8528 Japan; 5 Delta Kogyo Co. Ltd., 1-14 Shinchi, Fuchu-Cho, Aki-Gun, Hiroshima, 735-8501 Japan; 6 Department of Mechanical Engineering, The University of Tokyo, 7-3-1 Hongo, Bunkyo-Ku, Tokyo 113-8656 Japan; 7 Graduate School of Biomedical and Health Sciences, Hiroshima University, 1-2-3 Kasumi, Minami-Ku, Hiroshima, Hiroshima, 734-8553 Japan

**Keywords:** Prognosis, Biomedical engineering

## Abstract

In this paper, we propose a novel method for predicting acute clinical deterioration triggered by hypotension, ventricular fibrillation, and an undiagnosed multiple disease condition using biological signals, such as heart rate, RR interval, and blood pressure. Efforts trying to predict such acute clinical deterioration events have received much attention from researchers lately, but most of them are targeted to a single symptom. The distinctive feature of the proposed method is that the occurrence of the event is manifested as a probability by applying a recurrent probabilistic neural network, which is embedded with a hidden Markov model and a Gaussian mixture model. Additionally, its machine learning scheme allows it to learn from the sample data and apply it to a wide range of symptoms. The performance of the proposed method was tested using a dataset provided by Physionet and the University of Tokyo Hospital. The results show that the proposed method has a prediction accuracy of 92.5% for patients with acute hypotension and can predict the occurrence of ventricular fibrillation 5 min before it occurs with an accuracy of 82.5%. In addition, a multiple disease condition can be predicted 7 min before they occur, with an accuracy of over 90%.

## Introduction

Biometric information monitoring devices are used in various clinical scenarios such as surgeries and the intensive care units (ICUs)^1^. Many of these devices raise an alarm when clinical deterioration of the patient (detected via biological indices) is detected. For example, a pulse oximeter, which is capable of measuring the saturation of peripheral oxygen ($\hbox {SpO}_2$) through a simple pinch on the fingertip, raises an alarm when $\hbox {SpO}_2$ is below the threshold value (Generally 89–92%^2^). In other cases, blood pressure (e.g., diastolic blood pressure) can be continuously measured using a sphygmomanometer, and an alert is sounded when it falls below a set threshold. The thresholds for many of these alarms are set based on prior experiences of the healthcare provider and the patient’s condition^3^. These medical devices can perform long-term monitoring of the patients’ biological information and are important for efficient and effective treatment. However, conventional medical devices raise an alarm only after detecting a deterioration. This proves to be problematic for the medical staff, who cannot stay near the patient all the time.

To solve this problem, several studies have proposed clinical deterioration prediction systems^4,5^. For example, Langley et al.^4^ focused on the change of heart rate intervals and they proposed an approach to predict the development of idiopathic atrial fibrillation with an accuracy of 56.0%. This approach used deviance from the average heart rate interval as a predictor. Lynn and Chiang^6^ proposed an algorithm based on nonlinear features computed from the return and difference maps of the heart rate variability (HRV) signal and reported a sensitivity of 64%. In addition, Boon et al.^7^ predicted atrial fibrillation with 79.3% accuracy by using a support vector machine with the features extracted via a genetic algorithm among HRV parameters as predictors. Maryam et al.^8^ reported a sensitivity of 96.3% for predictions made using nonlinear features obtained from spectral analysis of the HRV signal. Wollmann et al.^9^ proposed a method that could predict ventricular arrhythmia with an accuracy of 72.5% by analysing HRV parameters calculated using an electrocardiogram by the classification and regression tree method.

To broaden the application range to other symptoms, methods that use machine learning^5,10,11^ were recently proposed. These methods can adapt the prediction model to the patients using the learning dataset. For example, in^10,11^, *k*-nearest neighbour and support vector machine were employed to predict acute hypotension, which is a type of shock symptom. However, these techniques did not consider the time series characteristics of the measured biological signals. This could be a reason why the prediction accuracy of these techniques was less than 90%. Henriques et al.^5^ employed a general regression neural network^12^ to predict acute hypotension. This prediction method received recognition as it achieved the highest prediction accuracy (92.5%) in Physionet Challenge 2009^13^. To represent the uncertainty in classification, these approaches calculate normalised indices in the range of [0, 1] from the outputs of the classifiers; however, they do not account for the probabilistic process of transition in physiological conditions.

Thus, we propose a target symptoms prediction method involving a probabilistic neural network called the recurrent log-linearised Gaussian mixture network (R-LLGMN)^14^. The R-LLGMN embeds a hidden Markov model (HMM) with multidimensional mixed Gaussian distribution, which are often used for time-series analysis and probabilistic classification, respectively. Because the parameters of HMM and Gaussian mixture model (GMM) are unified into connective weights through a log-linearisation process under the framework of a neural network, the R-LLGMN allows training the connective parameters comprehensively. This feature makes the proposed method suitable for probabilistic classification of time-series data^15–20^. In addition, using GMM to approximate the probability distribution is advantageous when dealing with a small dataset. This feature is important in clinical settings where a large dataset is not always available. In this paper, the target symptoms were acute hypotension and ventricular fibrillation (Vf) in addition to a multiple disease condition, and the prediction accuracy was tested using the data provided by Physionet^13^ and those collected from the University of Tokyo Hospital.

**Figure 1:**
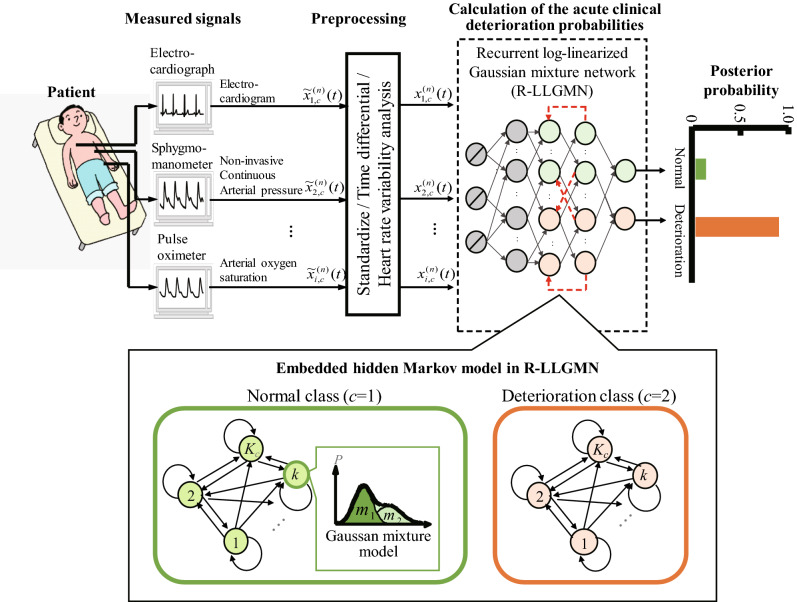
Overview of the proposed short-term prediction method.

## Materials and methods

### Proposed method

Figure 1 shows the proposed prediction method. First, the measured biological signals were preprocessed. Preprocessing includes calculation of indices related to HRV. The preprocessed signal was then fed to a probabilistic neural network (i.e., R-LLGMN) to predict probabilities of conditions in future *P* minutes. In this section, the proposed method is discussed in detail.

#### Preprocessing

An analysis on HRV was performed on the heart rate interval (RRI) acquired from an electrocardiograph. In this paper, we configured the RR recording interval as 1 minute in accordance with the previous studies^21–23^ on the short-term and ultra-short-term HRV analyses. The related indices include coefficient of variation of R-R intervals (CVRR) and the following indices that reflect vagal tone intensity^24,25^: root mean square successive difference^26^ (RMSSD) and number of pairs of successive RRI that differ by more than 50 [ms]^27^(pNN50). The aforementioned indices can be calculated using the following equations:

1 $$\begin{aligned} CVRR= & {} \frac{\sqrt{\frac{1}{N_{RRI}-1}\sum ^{N_{RRI}}_{i=1} \{RRI_{(i)}-RRI_{mean}\}^2}}{RRI_{mean}} \end{aligned}$$

2 $$\begin{aligned} RMSSD= & {} \sqrt{\frac{1}{N_{RRI}-1}\sum ^{N_{RRI}}_{i=1}\{RRI_{(i+1)}-RRI_{(i)}\}^2} \end{aligned}$$

3 $$\begin{aligned} pNN50= & {} \frac{N_{dif}50}{{N_{dif}}}, \end{aligned}$$

where $N_{RRI}$ is the total number of RRIs in 30 s, $RRI_{mean}$ is the average value of the RRIs in 30 s, $N_{dif}$ is total number of successive adjacent RRI differences, and ${N_{dif}50}$ is total number of successive adjacent RRI differences whose absolute values is greater than or equal to 50 [ms].

For biological signals that were not obtained from electrocardiographs, we employed the following preprocessing methods:

- Standardise the biological signal to the normal distribution $N(0, \sigma _d)$:
- Time-differentiation using a differential filter that can reduce measurement noise based on the centred difference method as follows^28^ :
  4 $$\begin{aligned} \Delta s(t) = \frac{1}{18 \Delta h} \left\{ \sum _{n=2}^4 s(t+n\Delta h) \right\} - \left\{ \sum _{n=2}^4 s(t-n\Delta h) \right\} , \end{aligned}$$
  where *s*(*t*) represents a measured biological signal and $\Delta h$ is the sampling time.

#### Proposed prediction model

Prediction model for acute deterioration triggered by the target symptoms must satisfy the following requirements:

1. Ability to account for the time series characteristics of biological signals.
2. Ability to express diversity of patients’ conditions.
3. Ability to express uncertainty of the predicted physical condition in a probabilistic manner.
4. Ability to simultaneously evaluate multiple types of biological signals.
5. Applicability to different medical fronts and different patients.

To satisfy the first two requirements, we apply HMM^29^. HMM can express various symptoms of the patient by applying a concept called “states” and “probabilistic transitions” between the states. For example, the condition of a patient can be defined as either “normal deterioration” or “acute deterioration”, and the temporal change of the biological signal drives the probabilistic transition between the defined conditions. However, biological signals are expected to be complex nonlinear waveforms. Therefore, they are approximated using multidimensional mixed Gaussian distribution model^30^, which is capable of expressing multimodal distribution by weighted summation of multiple Gaussian distributions^31^. To approximate complex waveforms of biological signals and to satisfy requirements (3) and (4), the proposed model was constructed based on continuous density HMM^32^, which is a combination of HMM and the multi-dimensional Gaussian mixture model. Probabilistic output of each state of the HMM can thus be represented by multi-dimensional mixed Gaussian distribution, which enables calculation of the occurrence probability of a deterioration event from multiple types of biological signals. The bottom row of Fig. 1 shows a physical change model represented by probability density distribution of the continuous multi-dimensional Gaussian model. Here, let us denote the number of evaluation target classes as $(c\in \{1 ,\ldots ,C\})$. Each class *c* is composed of $K_c$ states, indexed as $k, k' =1 , \ldots , K_c$, and the probability distribution of each state *k* includes $M_{c,k}$ Gaussian distribution components, indexed as $m = 1 , \ldots ,M_{c,k}$. The probability distribution of class *c* is represented by multidimensional Gaussian mixture distribution with $M_{c,k}$ Gaussian components. Given a time series biological signal ${{\varvec{X}}}=[{{\varvec{x}}}{(1)}, {{\varvec{x}}}{(2)}, \ldots , {{\varvec{x}}}{(T)}]$, where ${{\varvec{x}}}(t) \in \mathfrak {R}^d$, the a posteriori probability of class *c*, $P(c|{{\varvec{x}}}(t))$ is derived as follows:

5 $$\begin{aligned} P(c|{{\varvec{x}}}(t))= & {} \sum ^{K_c}_{k=1}P(c, k|{{\varvec{x}}}(t)) \end{aligned}$$

6 $$\begin{aligned} P(c, k|{{\varvec{x}}}(t))= & {} \frac{a^{c}_{k}(t)}{\sum ^C_{c'=1}\sum ^{K_{c'}}_{k'=1}a^{c'}_{k'}(t)} \end{aligned}$$

7 $$\begin{aligned} a^{c}_{k}(t)= & {} \sum ^{K_c}_{k'=1}a^{c}_{k'}(t-1)\gamma ^{c}_{k' ,k}b^{c}_{k}({{\varvec{x}}}(t))\qquad (t>1) \end{aligned}$$

8 $$\begin{aligned} a^{c}_{k}(1)= & {} \pi ^c_k b^c_k ({{\varvec{x}}}(1)) \end{aligned}$$

where $\gamma ^{c}_{k' ,k}$ is the probability of state transition from $k'$ to *k* in class *c*, and $b^{c}_{k}({{\varvec{x}}}(t))$ is defined as the a posteriori probability for state *k* in class *c* corresponding to ${{\varvec{x}}}(t)$. The prior probability $\pi ^c_k$ is equal to $P(c, k)|_{t=0}$.

Assuming that the posterior probability $b^{c}_{k}({{\varvec{x}}}(t))$ is given by a multidimensional Gaussian mixture model consisting of $M_{c ,k}$ components, $\gamma ^{c}_{k' ,k}b^{c}_{k}({{\varvec{x}}}(t))$ can be rewritten as follows:

9 $$\begin{aligned} \gamma ^{c}_{k' ,k}b^{c}_{k}({{\varvec{x}}}(t))= \sum ^{M_{c ,k}}_{m=1}\gamma ^{c}_{k' ,k}r_{(c ,k ,m)}g(x(t);\mu ^{(c ,k ,m)},\Sigma ^{(c ,k ,m)}), \end{aligned}$$

where $r_{(c ,k ,m)}$ is the mixing proportion, $\mu ^{(c ,k ,m)}\in \mathfrak {R}^d$ is the mean vector, and $\Sigma ^{(c ,k ,m)}\in \mathfrak {R}^{d\times d})$ is the covariance matrix of each component. The parameters included in the model are used to estimate the probability distribution and generate the posterior probability $P(c|{{\varvec{x}}}(t))$ of acute deterioration. To set a machine learning framework for the determination of parameters and to satisfy requirement (5), an R-LLGMN^14^ (see Supplementary Information S1) was employed.

The output of R-LLGMN is represented by the posterior probability of each class based on the multidimensional Gaussian mixture model. In addition, because the parameters of R-LLGMN can be adjusted for the given learning dataset, the proposed method can be applied to various symptoms. Let us represent a pair of learning data for R-LLGMN consisting of the input vector of ${\varvec{X}}^{(n)}_{c}=[{\varvec{x}}^{(n)}_{c}(1)$, ${\varvec{x}}^{(n)}_{c}(2), \ldots , {\varvec{x}}^{(n)}_{c}(t)$, $\ldots ,{\varvec{x}}^{(n)}_{c}(T_d)]$, where ${\varvec{x}}^{(n)}_{c}(t)=[x^{(n)}_{1, c}(t), \ldots , x^{(n)}_{i, c}(t), \ldots ,x^{(n)}_{I, c}(t)]^T$, and the corresponding teacher vector ${{\varvec{Y}}}^{(n)}=[Y^{(n)}_1, \ldots , Y^{(n)}_c,\ldots , Y^{(n)}_C]^{T}$. Here, the element $x_{i, c}{}^{(n)} (t)$ is a biological signal measured at time *t*, and $Y^{(n)}_c$ is the posterior probability of class *c*. Each index represents the following: $n=1, 2,\ldots N$ is the dataset number, $T_d$ is the total time step to output a posterior probability vector $O_c{}^{(n)}$ at the output layer of R-LLGMN, class $c=1$ represents the class for normal condition, and $c=2$ represents the class for occurrence of deterioration event, such that $C=2$. The evaluation function *J* is then defined by the following equation:

10 $$\begin{aligned} J=\sum ^N_{n=1}J_n=-\sum ^N_{n=1}\sum ^C_{c=1}Y^{(n)}_c\log ^{(5)}O^c(T)^{(n)} \end{aligned}$$

The learning process is applied to minimise the above function (i.e., maximising the likelihood). The weight parameters in R-LLGMN are iteratively updated using backpropagation through time^33^ (BPTT). BPTT is a method of accumulating the error gradient in the time series and calculating the weight correction amount for each iteration. After the parameters included in the first and second layer are adjusted, R-LLGMN can predict the class of the condition, such as normal or acute deterioration, of the target patients *P* minutes from the acquisition of the biological signals.

### Experimental configuration

**Table 1:** A list of datasets used in the experiments.

	Number of patients	Positives	Negatives	Type of disease	Provider
Dataset 1	60	30	30	Acute hypotension	Physionet
Dataset 2	40	14	26	Acute hypotension	Physionet
Dataset 3	20	20	20	Vfentricular fibrillation	Physionet
Dataset 4	15	39	30	A multiple disease condition (These name are hidden)	The University of Tokyo Hospital

In order to verify the prediction performance of the proposed model, a prediction experiment was conducted using datasets that include various cases of ICU patients, published by Physionet^13^ and the University of Tokyo Hospital. The dataset provided by the ICU at the University of Tokyo Hospital is composed of vital signs of patients in the ICU, with a sampling time of 1024 [ms]. In addition, two types of information are given as expert annotations. These are technical validity and clinical relevance of the alarm raised by the biological information monitor. Technical validity is the result of the diagnosis performed by the nurse when an alarm is raised, and clinical relevance is the result of the diagnosis performed by the doctor when the alarm is raised.

For the Physionet^13^ dataset, one of the authors took part in an online ethics training program called “protecting human research participants^34^” (Certification number: 1756830, Acquisition Date: May. 2, 2015). For the University of Tokyo Hospital dataset^35,36^, the authors confirmed that all data were provided with authorisation from the University of Tokyo Hospital ethics committee. Furthermore, informed consent was taken from all examinees or their families. Using the above datasets, the following five analyses were conducted:

(i) Preprocessing selection: The influence of two types of preprocessing methods on the prediction accuracy was tested to determine the best preprocessing method. In addition, the optimal $\sigma _d$ was also determined.
(ii) R-LLGMN hyperparameters selection: The influence of $M_{c,k}$ and $K_c$ on the prediction accuracy and the learning time was tested using the same dataset that was used for preprocessing selection.
(iii) Prediction of Acute hypotension: Using the hyperparameters determined above, the prediction accuracy on occurrence of acute hypotension was examined. The accuracy was compared with the previous methods.
(iv) Prediction of Vf: The accuracy of Vf prediction was tested. This analysis aims to investigate whether the proposed method can predict occurrence of acute diseases other than acute hypotension.
(v) Prediction of multiple symptoms: The prediction accuracy of events triggered by a multiple disease condition was tested.

In all experiments, the positive threshold was calculated by performing receiver operating characteristic (ROC) analysis on the learning dataset. In addition, we defined patients with deterioration as the patients whose events occurred within *P* minutes, corresponding to class $c=2$, and patients in normal condition as those patients whose events did not occur within *P* minutes, corresponding to class $c=1$. Next, the configurations are discussed in detail.

#### Preprocessing selection

Dataset 1 provided by Physionet (see Table 1) was used to determine the best preprocessing method. Dataset 1 has a total of 60 patients’ data, among which 30 patients had developed acute hypotension while the remaining 30 patients had not. The sampling time of Dataset 1 is 60 s, and the input biological signals are heart rate, systolic blood pressure, diastolic blood pressure, and mean blood pressure. The data was trimmed to 12 min based on preliminary experiment (see Supplementary information S3). Two types of processing, (a) normalisation processing and (b) time-differential processing, were performed to investigate the influence on accuracy. For preprocessing (a), $\sigma _d$ was varied as follows: $\sigma _d$ = 1, 0.1, 0.01, 0.001. In these analyses, the learning process of R-LLGMN was repeated five times with different initial weights, and the average prediction accuracy was calculated. Iterative two-way analysis of variance (ANOVA) was performed to compare methods (a) and (b). If interactions were confirmed with a significance level of less than 5[%], multiple tests based on the Bonferroni method were performed with $p < 0.05$ as the significance level. In statistical processing in (b), multiple tests based on the Bonferroni method were performed under a significance level of 5%. In addition, multiple tests based on the Bonferroni method were also performed to compare the effect between different $\sigma _d$ values. Three combinations of hyperparameters $M_{c,k}$ and $K_c$ included in R-LLGMN were set as: $(M_{c,k}, K_c)=(1, 2), (2, 3), (3, 3)$.

#### R-LLGMN hyperparameter selection

The influence of $M_{c,k}$ and $K_c$, on prediction accuracy and learning time was tested using Dataset 1. This experiment was performed only on the normalisation process. The hyperparameters were varied in ranges of $M_{c,k}=1, 2, 3, 4, 5$ and $K_c=1, 2, 3, 4, 5$. Hyperparameter $\sigma _d$ was set to be 0.01. Other hyperparameters’ settings were the same as those described in the previous section. The leave-one-patient-out cross-validation method was employed to calculate prediction accuracy and learning time. The CPU of the PC used in this experiment employed an Intel Xeon (R) (X5667: Intel Corporation, number of cores: 4, clocking frequency: 3.1 GHz), memory (16.0 GB, DDR 3 800/1066/1333).

#### Prediction of acute hypotension occurrence

To verify the prediction performance of the proposed model, a comparison with a previous prediction method was conducted. The learning dataset and configuration are the same as those used in R-LLGMN hyperparameter selection; however, only the normalisation preprocessing with $\sigma _d=0.01$ was performed. The test dataset used for verification was Dataset 2 (see Table 1), among which 14 patients had developed acute hypotension whereas the remaining 26 patients had not. The sampling time was 1 min; the input signals were heart rate, systolic blood pressure, diastolic blood pressure, and mean blood pressure. Among the previous methods published by Physionet Challenge^5^, the method by Henriques et al., the one which achieved the highest accuracy, was chosen as the comparison target^11^. To compare the proposed method under the same conditions as the previous method, prediction accuracy of acute hypotension, sensitivity, and specificity were calculated using the test data.

#### Prediction of Vf occurrence

Prediction of Vf was performed using Dataset 3 (see Table 1), which was provided by Physionet and composed of patients with Vf. Here, because Dataset 3 only contains the patients with positive events, we extracted a negative time span from each patient to constitute negative data such that the number of positive and negative data was equalised. Here, normalisation preprocessing was selected as the preprocessing method. First, the parameters related to RRI were calculated and the influence of input parameters on accuracy was investigated. The prediction accuracy for a one-dimensional input (input data: RRI) and a three-dimensional input (input data: CVRR, RMSSD, and pNN50) was compared. The influence of the prediction time *P* on the prediction accuracy was also investigated. Twenty patients with Vf participated for this analysis. As Vf occurred once for each patient, the normal data could be extracted from the same patient but different time spans. This resulted in 20 samples with occurrence of Vf and 20 samples under normal conditions. Here, the normal data was extracted from the data by excluding one hour before and after the occurrence of Vf. The sampling frequency was 250 [Hz]. The analysis target data was trimmed to 30 s. In preprocessing, $\sigma _d$ = 0.1 was used for one-dimensional inputs and $\sigma _d$ = 0.01 was used for three-dimensional inputs. Leave-one-event-out cross-validation method was applied to test the influence of prediction time *P* minutes ahead of the actual occurrence of Vf. Here, *P* was changed from 1 to 10 min in intervals of 1 min. The above procedure was repeated 10 times with different initial weights of R-LLGMN, and the average prediction accuracy was calculated. A statistical comparison was performed using the Welch test with a significance level of 5%.

#### Prediction of symptom events triggered by a multiple disease condition

Prediction of symptom events triggered by a multiple disease condition was performed using Dataset 4 (see Table 1). Dataset 4 contains biological signals such as heart rate and arterial blood pressure measured from ICU patients provided by Department of Emergency and Critical Care Medicine of the University of Tokyo Hospital^35,36^. In this experiment, patients whose blood pressure gauge alarms were confirmed to be clinically and technically appropriate were defined as patients with a symptom event for their respective disease. Other patients were defined as normal in this experiment. Among 15 patients, doctors confirmed that the blood pressure gauge raised the correct alarms for a total of 39 times in nine patients (Sub. A-I) and 30 times the false alarms in the other six patients (Sub. J-O). The number of confirmed symptom events for each patient is as follows: Sub. A: 15 times, Sub. B: 2 times, Sub. C: 5 times, Sub. D: 6 times, Sub. E: 2 times, Sub. F: 2 times, Sub. G: 1 time, Sub. H: 1 time, and Sub. I: 5 times. The time durations that include the symptom events obtained from Sub. A-I comprised 39 positive event data. The negative event data is composed of the five different time periods extracted from every six normal patients with false alarms (Sub. J-O). As such, 39 data of symptom events and 30 data under normal conditions were obtained (see Table 1).

The prediction accuracy was calculated using leave-one-event-out cross-validation with different *P*; *P* was changed from 1 to 7 min with 1 min intervals. Here, normalisation preprocessing was selected as the preprocessing method. The input signals were heart rate, systolic blood pressure, diastolic blood pressure, and mean blood pressure. ${\sigma}_{\mathrm{d}} = 0.01$, and the analysis period was 30 s.

**Table 2:** Interaction between time-differential processing and normalisation processing for hyperparameters $M_{c,k}$ and $K_c$.

Source	Sum of squares	Degrees of freedom	Mean squares	Variance ratio *F*	*p*-value
I. $M_{c,k}$ and $K_c$ are set 1 and 2					
Differential processing	0	1	0	0	1.0
Normalisation processing	802.2	1	802.2	186.3	3.1$$\times 10^{-10 **}$$
Interaction	142.2	1	142.2	33.0	3.0$$\times 10^{-5 **}$$
Error	68.8	16	4.3		
Total	1013.3	19			
II. $M_{c,k}$ and $K_c$ are set 2 and 3					
Differential processing	347.2	1	347.2	48.5	3.2$$\times 10^{-6 **}$$
Normalisation processing	20.0	1	20.0	2.8	1.1$\times 10^{-1}$
Interaction	293.9	1	293.9	41.1	8.6$$\times 10^{-6 **}$$
Error	114.4	16	7.2		
Total	775.6	19			
III. $M_{c,k}$ and $K_c$ are set 3 and 3					
Differential processing	50.1	1	50.1	7.5	$1.4\times 10^{-2 *}$
Normalisation processing	101.3	1	101.3	15.2	$$1.3\times 10^{-3 **}$$
Interaction	133.5	1	133.5	20.0	$$3.8\times 10^{-4 **}$$
Error	106.7	16	6.7		
Total	391.5	19			

$$* : p<0.05\quad ** : p<0.01$$

**Figure 2:**
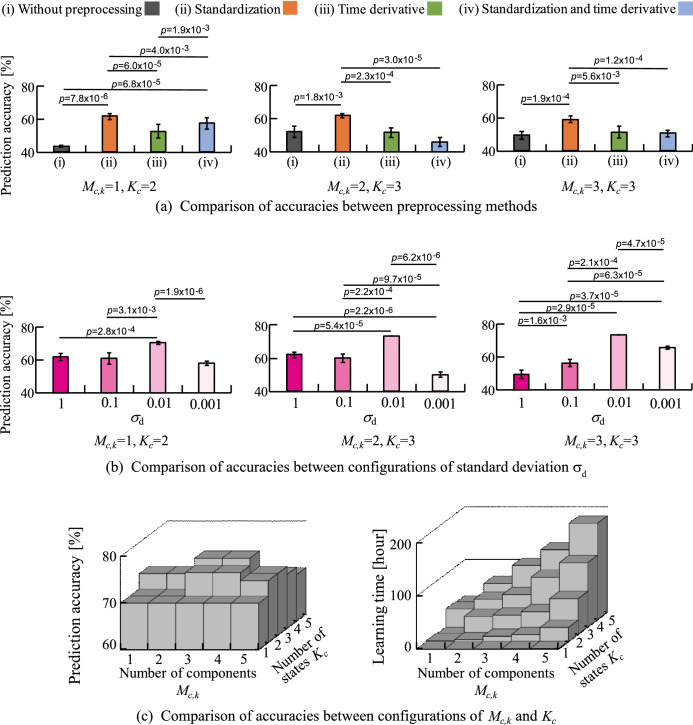
Comparison of accuracies for different configurations. (**a**) Compares the preprocessing methods. (**b**) Compares the different values of standard deviation parameter $\sigma _d$ (hyperparameter). Both comparisons were carried out by setting $(M_{c,k}, K_c)=(1,1), (2,3), (3,3)$. (**c**) Accuracies and the required time duration for learning in different configurations of hyperparameters $M_{c,k}$ and $K_c$.

## Results

### Preprocessing selection

The results for selection of preprocessing method are shown in Table 2. The table shows a two-way ANOVA for hyperparameters $(M_{c,k}, K_c)=(1, 2), (2, 3), (3, 3)$. Based on Table 2. I, it is not confirmed that there was a significant difference in the influence of time-differential processing on the prediction accuracy ($p=1.0$). In contrast, the normalisation process was confirmed to have a significant effect on the prediction accuracy. Moreover, a significant interaction between time-differential preprocessing and normalisation preprocessing was confirmed ($p=3.1 \times 10^{-10}, p=3.0 \times 10^{-5}$, respectively). Based on Table 2. II, a significant difference in the effect of time-differential preprocessing on the accuracy ($p=3.2 \times 10^{-6}$) was observed. It was also confirmed that there was a significant interaction between time-differential preprocessing and normalisation preprocessing ($p=8.6 \times 10^{-6}$). However, a significant difference in the influence of normalisation preprocessing on accuracy ($p=1.1 \times 10^{-1}$) was not confirmed. Based on Table 2. III, it was confirmed that there was a significant difference in the influence of differentiation preprocessing and normalisation preprocessing on prediction accuracy ($p=1.4 \times 10^{-2}, p=1.3 \times 10^{-3}$, respectively). In addition, it was confirmed that there was a significant interaction between time-differential preprocessing and normalisation preprocessing ($p=3.8 \times 10^{-4}$). The above results show that time-differential preprocessing and normalisation preprocessing affect each other for all $(M_{c,k}, K_c)=(1, 2), (2, 3), (3, 3)$. Therefore, the four groups can be regarded as independent groups and multiple tests based on the Bonferroni-adjusted method were performed for a significance level of 5%.

Figure 2a shows the average prediction accuracy calculated using the following four different preprocessing: (i) Without preprocessing, (ii) normalisation preprocessing, (iii) time-differential preprocessing, and (iv) time-differential and normalisation preprocessing. From Fig. 2a, when $M_{c,k}=1$, $K_c=2$, the accuracy and standard deviation for (i), (ii), (iii), and (iv) are $43.7 \pm 0.7\%,61.7 \pm 2.0\%,49.0 \pm 2.8\%,$ and $56.3 \pm 2.2\%$, respectively. In addition, it is confirmed that there was a significant difference between all the groups, except between (i) and (iii) and between (i) and (ii). When $M_{c,k}=2$, $K_c=3$, the accuracy and standard deviation for (i), (ii), (iii), and (iv) were $52.3 \pm 3.4\%,62 \pm 1.4\%, 51.7 \pm 2.6\%,$ and $46.0 \pm 2.8\%$, respectively. Significant differences were confirmed between (i) and (ii), (ii) and (iii), and (ii) and (iv). When $M_{c,k}=3$, $K_c=3$, the accuracy and standard deviation for (i), (ii), (iii), and (iv) are $49.3 \pm 2.5\%, 59.0 \pm 1.9\%, 51.3 \pm 3.6\%,$ and $50.7 \pm 1.9\%$, respectively. In addition, significant difference was confirmed among (i) and (ii), (ii) and (iii), and (ii) and (iv). From these results, it can be determined that the accuracy was highest when normalisation preprocessing is performed under the conditions of $(M_ {c, k}, K_c)=(1, 2), (2, 3), (3, 3)$. It was also confirmed that there was a significant difference between all groups.

Figure 2b shows the average prediction accuracy for different $\sigma _d$. When $M_{c,k}=1$, $K_c=2$, the prediction accuracy for $\sigma _d = 1, 0.1, 0.01$, and 0.001 were $61.7 \pm 2.0\%, 61.0 \pm 3.5\%, 70.3 \pm 0.7\%, 58 \pm 1.3\%$, respectively. A significant difference was confirmed between $\sigma _d = 1$ and $\sigma _d = 0.01$, $\sigma _d = 0.1$ and $\sigma _d = 0.01$, and $\sigma _d = 0.01$ and $\sigma _d = 0.001$. When $M_{c,k}=2$, $K_c=3$, the prediction accuracies were $62.0 \pm 1.4\%, 60.0 \pm 2.4\%, 73.3 \pm 0.0\%, 50.0 \pm 1.7\%$, respectively. A significant difference was confirmed between all the groups, except between $\sigma _d = 1$ and $\sigma _d = 0.1$. When $M_{c,k}=3$,$K_c=3$, average prediction accuracies and standard deviations were $49.3 \pm 2.5\%, 56.0 \pm 2.1\%, 73.3 \pm 0.0\%, and 65.0 \pm 0.9\%$. In addition, it was confirmed that there was a significant difference between all groups. Therefore, the accuracy was highest when $\sigma _d= 0.01$ and a significant difference between all groups in the conditions of $(M_ {c, k}, K_c) = (1, 2), (2, 3), (3, 3)$ was confirmed.

### R-LLGMN hyperparameter selection

Figure 2c shows the prediction accuracy and time required for learning when $M_ {c, k}$, $K_c$ is varied in the range of 1–5. The prediction accuracy was improved with an increase in $M_{c,k}$ and $K_c$. The prediction accuracy was maximised (76.6 %) when $M_{c,k}=3$,$K_c=3$ and $M_{c,k}=3$,$K_c=4$. It then decreased as $M_ {c, k}$ and $K_c$ increased. In addition, it was confirmed that time required for learning increases with the increase in $M_ {c, k}$ and $K_c$.

**Figure 3:**
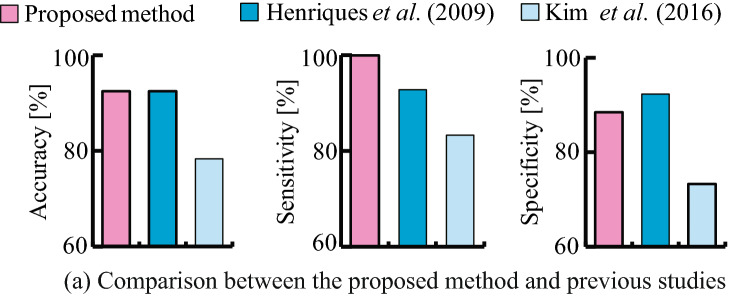
Prediction accuracy of the proposed method. The figure compares prediction accuracy between the proposed method and the previous methods^5,11^.

#### Prediction of acute hypotension occurrence

The data from a total of 60 patients was extracted from Physionet^13^ (see section “Experimental configuration”) and used to test prediction accuracy for acute hypotension. We compared the prediction accuracy of the proposed method against some previous studies^5,11^ in which Physionet datasets^13^ were also used. Figure 3 shows the results of comparison of the prediction accuracies between the proposed method and the previous methods. From the figure, it can be seen that the accuracy, sensitivity, and specificity of the proposed model were 92.5%, 100.0%, and 88.5%, respectively. A receiver operation characteristic analysis confirmed the area under the curve (AUC) value of 0.86. From this result, the proposed model had the same prediction accuracy (92.5%) as the method proposed by Henriques et al., which achieved the highest accuracy among the methods published in Physionet Challenge 2009^13^. Moreover, it was also confirmed that the proposed method has higher sensitivity (100.0%) than some of the previous methods.

**Figure 4:**
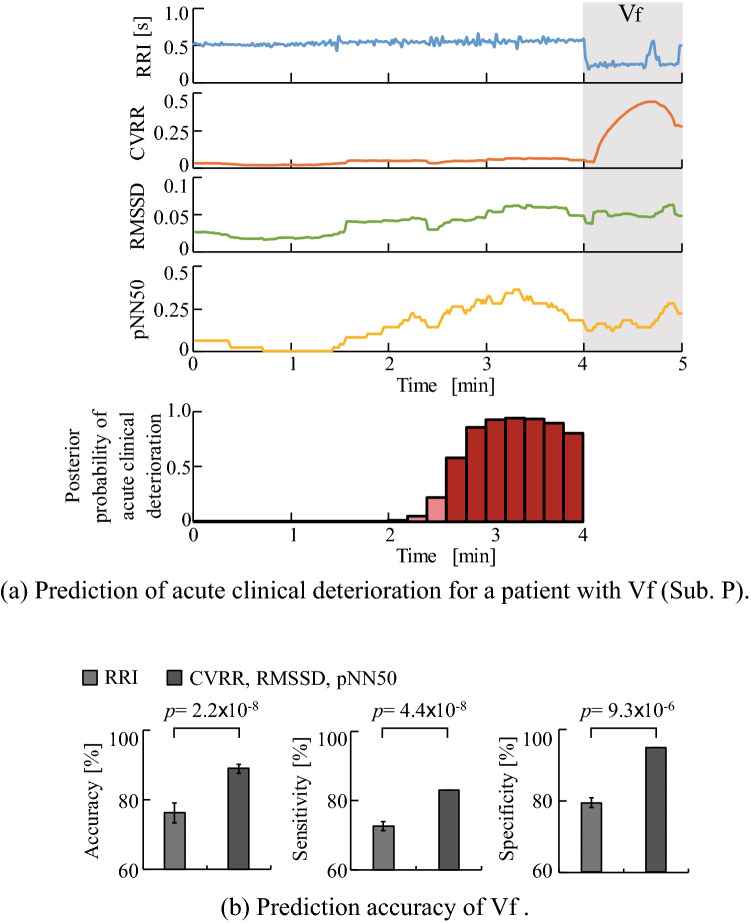
Prediction of acute clinical deterioration triggered by Vf. (**a**) Time course change in HRV indices and the posterior probability of acute clinical deterioration triggered by Vf (Sub.P) calculated by the proposed method. The grey highlight indicates the occurrence of Vf. (**b**) Compares prediction accuracies between different input dimensions in terms of average accuracy, sensitivity, and specificity. The blue bar indicates accuracy achieved when RRI was used as the input. The red bar indicates accuracy achieved when CVRR, RMSSD, and pNN50 were together used as the input.

**Table 3:** Confusion matrix and accuracies of patients with Vf for different prediction time points *P*.

Prediction time *P*	1	2	3	4	5	6	7	8	9	10
True positive	17	17	16	14	15	14	12	12	13	9
True negative	19	19	19	19	18	17	17	17	17	17
False negative	3	3	4	6	5	6	8	8	7	11
False positive	1	1	1	1	2	3	3	3	3	3
Accuracies [%]	90.0	90.0	87.5	82.5	82.5	77.5	72.5	72.5	75.0	65.0

#### Prediction of Vf occurrence

Dataset 3 provided by Physionet^13^ is used to test the prediction accuracy of Vf (see section “Experimental configuration”). Figure 4a shows the time series posterior probabilities of Vf occurrence of a patient (Sub. P) when CVRR, RMSSD, and pNN50 were together used as inputs for each 10 s period. The figure confirms that posterior probabilities increase as a function of time till the occurrence of Vf reduces. Figure 4b compares accuracy, sensitivity, and specificity between a one-dimensional and a three-dimensional input. The prediction time was set to $P=1$ minute. The figure confirms that there is a significant increase in accuracy, sensitivity, and specificity for the three-dimensional input compared to a one-dimensional input. Therefore, it was confirmed that the prediction accuracy improves when using multidimensional inputs. Table 3 shows the confusion matrix and prediction accuracies for all patients with different prediction time points *P* minutes ahead of the occurrence of Vf. Based on Table 3, prediction accuracies at prediction time points *P*=1, 2, ...,10 are 90.0%, 90.0%, 87.5%, 82.5%, 82.5%, 77.5%, 72.5%, 72.5%, 75.0%, and 65.0%, respectively. The AUC values at prediction time points *P*=1, 2, ...,10 are 0.94, 0.94, 0.85, 0.91, 0.90, 0.84, 0.70, 0.74, 0.72, and 0.62, respectively. Therefore, the prediction accuracy increases as we approach the time of occurrence of Vf.

**Figure 5:**
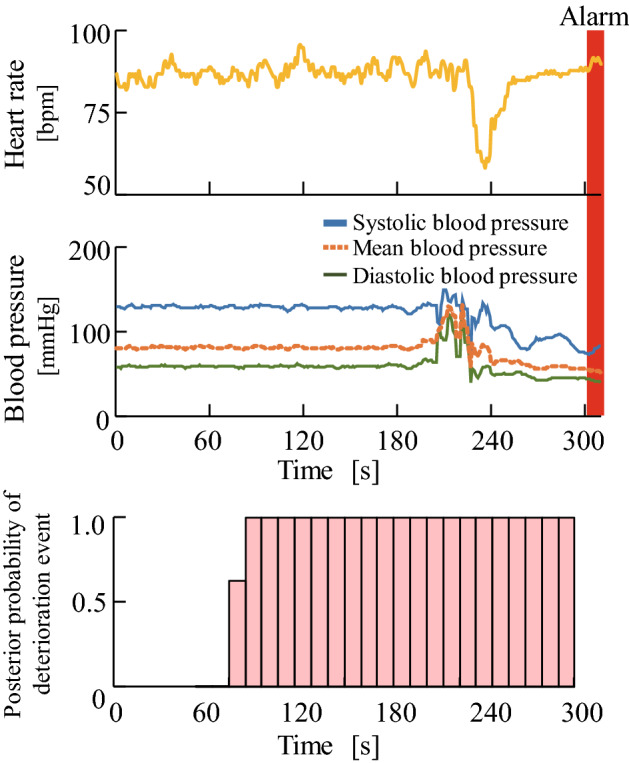
Analysis results of heart rate, arterial pressure, and prediction of acute clinical deterioration for a patient with a multiple disease condition (Sub. A).

**Table 4:** Confusion matrix and identification rates of patients with a multiple disease condition for different prediction time points *P*.

Prediction time *P*	1	2	3	4	5	6	7
True positive	37	36	36	35	34	36	34
True negative	30	29	30	30	29	28	29
False negative	2	3	3	4	5	3	4
False positive	0	1	0	0	1	2	1
Accuracies [%]	97.1	94.2	95.7	94.2	91.3	92.8	91.3

#### Prediction of symptom events triggered by a multiple disease condition

The dataset provided by the ICU at the University of Tokyo Hospital was used to test the prediction accuracy of symptom events triggered by an undiagnosed multiple disease condition (see section “Experimental configuration”). Figure 5 shows the time-series posterior probabilities of a symptom event in a patient (Sub. A) when measured biological signals were input for each 10 s period. The figure confirms that the posterior probabilities increase as time approaches the symptom event. Table 4 shows the confusion matrix and prediction accuracies for all patients from *P* = 1 min to *P* = 10 min. The prediction accuracies at prediction time points *P*=1, 2, ..., 7 are 97.1%, 94.2%, 95.7%, 94.2%, 91.3%, 92.8%, and 91.3%, respectively. The AUC values at prediction time points *P*=1, 2, ..., 7 are 0.98, 0.97, 0.94, 0.94, 0.95, 0.93, and 0.94, respectively. Therefore, the prediction accuracy increases as we approach the time of occurrence of acute clinical deterioration.

## Discussion

With the aim of predicting an acute deterioration triggered by target symptoms, we proposed a prediction method employing a probabilistic neural network that embeds the hidden Markov model with multidimensional mixed Gaussian distribution, called R-LLGMN. It enables prediction of a symptom event from multiple biological signals using the probability transition process in physiological conditions. The parameters of the model can be acquired through machine learning; hence, it can potentially be applied to various symptoms.

To determine the appropriate preprocessing method and model configuration, we statistically analysed the prediction accuracies generated under different settings using the data provided by Physionet. We then found that the prediction accuracy peaks when normalisation preprocessing is performed (see Fig. 2a). This is because appropriate scaling of the input data eliminates the difference in amplitude between data, which is irrelevant for R-LLGMN to discriminate between the two classes. Significant differences between time-differential preprocessing and no preprocessing was not confirmed when the hyperparameters in R-LLGMN were set to the following values: $(M_{c,k},K_c)=(1, 2), (2, 3), (3, 3)$ (see Fig. 2a). This is because time-differential preprocessing can only represent information on short-term temporal changes in the biological signal, making it difficult to make long-term predictions. These results indicated that normalisation is an effective preprocessing that enabled to obtain the highest accuracy. In addition, we confirmed that the prediction accuracy was the highest when $\sigma _d=0.01$ (see Fig. 2b). This is because variation in the input data affected the learning of R-LLGMN. These results indicate that hyperparameter $\sigma _d$ must be determined based on the variation in the input data used for prediction. Therefore, in the following analysis, a preliminary analysis was conducted to determine $\sigma _d$. However, a detailed investigation on the method for selecting $\sigma _d$ will be necessary in the future.

In terms of the neural network configuration, it was demonstrated that the prediction accuracy becomes maximum when $M_{c,k}=3$, $K_c=3$ and $M_{c,k}=3$, $K_c=4$ (see Fig. 2c). This is because increasing $M_{c,k}$ and $K_c$ enables R-LLGMN to model complicated time series characteristics by improving its representation ability. Moreover, it was demonstrated that prediction accuracy decreases when the values of the hyperparameters are increased to more than $M_{c,k}=3$, $K_c=3$ and $M_{c,k}=3$, $K_c=4$. This is due to overfitting, which can worsen the generalisation performance. In addition, $M_{c,k}$, $K_c$ increases the time required for the learning process (see Fig. 2c) because the computational complexity increases. Therefore, considering the trade-off between learning time and prediction accuracy, $M_{c,k}=3$, $K_c=3$ ($c=1, 2$, $k=1, 2, 3$) were considered as the optimal hyperparameters.

Based on the hyperparameters and model configuration, the prediction accuracies were tested for acute hypotension, Vf, and a multiple disease condition. The prediction results for acute hypotension confirmed that the proposed model has the same level of prediction accuracy (92.5%) and sensitivity (100%) as the method proposed by Henriques et al. (see Fig. 3).

The prediction results for Vf confirmed a significant increase in accuracy, sensitivity, and specificity when using a three-dimensional input (see Fig. 4b). This is because not only the time series characteristics of RRI, but also the vagus nerve activity of the patient could be evaluated. Vagal nerve activity has been reported to increase or decrease^37^ before the onset of Vf. Thus, it is effective in predicting Vf, which is a type of ventricular arrhythmia. In addition, an increase in the number of input dimensions also contributed to an improvement in prediction accuracy as it enabled R-LLGMN to extract characteristics of multiple types of biological information. However, the prediction accuracy decreased as the prediction time point parameter *P* increased. This indicates difficulties in early prediction (see Table 3). Introducing frequency analysis on the biological signals and applying it as an additional input dimension may improve early prediction accuracy.

The prediction result for a multiple disease condition confirmed that the posteriori probability increases as the prediction time point *P* approaches the point of a symptom event (see Fig. 5). Table 4 shows that all the prediction accuracies at prediction time points from $P=1$ min to $P=7$ min before the occurrence of a symptom event exceed 90.0%. This verifies the effectiveness of the proposed method in predicting events triggered by a multiple disease condition.

In this paper, we only tested prediction accuracy using a limited number of combinations of hyperparameters ($K_c$ and $M_{c,k}$). Testing with more combinations could provide better prediction accuracy or enable earlier detection. In addition, optimising the duration of RR interval analysis may also contribute to better performance. However, searching the combinations of hyperparameters is considerably time-consuming, and large values of $K_c$ and $M_{c,k}$ may cause overlearning. A more efficient learning algorithm is required to optimise the hyperparameters for the neural networks used in this paper.

All input indices employed in this study are the linear variables in the time domain, but linear and nonlinear variables in the frequency domain such as standard deviation of HRV and power of HRV in the high- and low-frequency bands are reportedly more effective for predictive clinical purposes^23,38–40^. However, the electrocardiogram data used in this study were sampled at 250 Hz, which was insufficient to estimate the frequency information of the heart rate interval accurately. Further improvement of the prediction accuracies and earlier detection of deterioration may thus be achieved by incorporating the frequency domain indices derived from the electrocardiogram data sampled at higher sampling frequencies. It should be noted that when adding these indices as the input features, the proposed method does not need to change its fundamental structure and algorithm because it adopts an R-LLGMN-based machine learning framework.

The number of patients analysed is not ideal. The database we used (Physionet Challenge 2009) only provides data for 30 patients with acute clinical deterioration for the learning dataset and 14 patients with acute clinical deterioration for the test dataset. Although we analysed four different datasets using the proposed algorithm with a single network architecture and the results demonstrated in this paper indicate the success and versatility of the proposed method, it is necessary to increase the number of patients from other open databases such as MIMIC III to further enhance the generalisability of the proposed method.

The results of our experiments showed that the proposed model has the highest prediction accuracy compared to contemporary methods. In addition, the proposed method is capable of predicting a symptom event triggered by different diseases, such as acute hypotension and Vf, by adjusting the parameters of the model using the corresponding learning data. Given that the proposed method can predict a target symptom event before it actually occurs with a high accuracy of approximately 90%, we can conclude that the proposed method has achieved a clinically applicable precision.

## Supplementary information

Supplementary information.

## References

[1] Ministry of Health, Labor and Welfare Summary of Patient Survey, howpublished. http://www.mhlw.go.jp/english/database/db-hss/sps_2014.html (2014). Accessed 13 Feb 2017.

[2] Kohyama T (2015). Accuracy of pulse oximeters in detecting hypoxemia in patients with chronic thromboembolic pulmonary hypertension. PLoS ONE.

[3] Schmid F (2011). The wolf is crying in the operating room patient monitor and anesthesia workstation alarming patterns during cardiac surgery. Anesth. Analg..

[4] Langley, P. *et al.* Can paroxysmal atrial fibrillation be predicted? in *Computers in Cardiology 2001*, 121–124 (IEEE, 2001).

[5] Henriques, J. & Rocha, T. Prediction of acute hypotensive episodes using neural network multi-models. in *Computers in Cardiology, 2009*, 549–552 (IEEE, 2009). 10.1016/j.compbiomed.2011.07.006 21899833

[6] Lynn, K. & Chiang, H. A two-stage solution algorithm for paroxysmal atrial fibrillation prediction. in *Computers in Cardiology*, 405–407 (IEEE, 2001).

[7] Boon K, Khalil-Hani M, Malarvili M, Sia C (2016). Vparoxysmal atrial fibrillation prediction method with shorter hrv sequences. Comput. Methods Programs Biomed..

[8] Mohebbi M, Ghassemian H (2012). Prediction of paroxysmal atrial fibrillation based on non-linear analysis and spectrum and bispectrum features of the heart rate variability signal. Comput. Methods Programs Biomed..

[9] Wollmann C (2015). Variations of heart rate variability parameters prior to the onset of ventricular tachyarrhythmia and sinus tachycardia in icd patients. results from the heart rate variability analysis with automated icds (hawai) registry. Physiol. Meas..

[10] Fournier, P. & Roy, J. Acute hypotension episode prediction using information divergence for feature selection, and non-parametric methods for classification. in *Computers in Cardiology, 2009*, 625–628 (IEEE, 2009).

[11] Kim S-H, Li L, Faloutsos C, Yang H-J, Lee S-W (2016). Heartcast: Predicting acute hypotensive episodes in intensive care units. Stat. Methodol..

[12] Specht DF (1991). A general regression neural network. IEEE Trans. Neural Networks.

[13] A challenge from PhysioNet, howpublished. https://physionet.org/challenge/2009/ (2009). Accessed 01 Sep 2015.

[14] Tsuji T, Bu N, Fukuda O, Kaneko M (2003). A recurrent log-linearized gaussian mixture network. IEEE Trans. Neural Netw..

[15] Tsuji T, Fukuda O, Ichinobe H, Kaneko M (1999). A log-linearized gaussian mixture network and its application to eeg pattern classification. IEEE Trans. Syst. Man Cybern. Part C (Appl. Rev.).

[16] Bu N, Okamoto M, Tsuji T (2009). A hybrid motion classification approach for emg-based human-robot interfaces using bayesian and neural networks. IEEE Trans. Rob..

[17] Takaki T (2015). Electromyographic prosthetic hand using grasping-force-magnification mechanism with five independently driven fingers. Adv. Robot..

[18] Hayashi H, Shibanoki T, Shima K, Kurita Y, Tsuji T (2015). A recurrent probabilistic neural network with dimensionality reduction based on time-series discriminant component analysis. IEEE Trans. Neural Netw. Learn. Syst..

[19] Soh Z, Kitayama S, Hirano A, Tsuji T (2013). Bioassay system based on behavioral analysis and bioelectric ventilatory signals of a small fish. IEEE Trans. Instrum. Meas..

[20] Furui A (2019). A myoelectric prosthetic hand with muscle synergy–based motion determination and impedance model–based biomimetic control. Sci. Robot..

[21] Salahuddin, L., Cho, J., Jeong, M.G. & Kim, D. Ultra short term analysis of heart rate variability for monitoring mental stress in mobile settings. in *2007 29th annual international conference of the ieee engineering in medicine and biology society*, 4656–4659 (IEEE, 2007). 10.1109/IEMBS.2007.4353378 18003044

[22] Baek HJ, Cho C-H, Cho J, Woo J-M (2015). Reliability of ultra-short-term analysis as a surrogate of standard 5-min analysis of heart rate variability. Telemed. e-Health.

[23] Nussinovitch U (2011). Reliability of ultra-short ecg indices for heart rate variability. Ann. Noninvas. Electrocardiol..

[24] Koelstra S (2012). Deap: A database for emotion analysis; using physiological signals. IEEE Trans. Affect. Comput..

[25] Molfino A (2009). Body mass index is related to autonomic nervous system activity as measured by heart rate variability. Eur. J. Clin. Nutr..

[26] Force T (1996). Standards of measurement, physiological interpretation and clinical use. task force of the european society of cardiology and the north american society of pacing and electrophysiology. Circulation.

[27] Bigger JT (1988). Components of heart rate variability measured during healing of acute myocardial infarction. Am. J. Cardiol..

[28] Usui S, Amidror I (1982). Digital low-pass differentiation for biological signal processing. IEEE Trans. Biomed. Eng..

[29] Seymore, K., McCallum, A. & Rosenfeld, R. Learning hidden markov model structure for information extraction. in *AAAI-99 workshop on machine learning for information extraction*, 37–42 (1999).

[30] Everitt B (1996). An introduction to finite mixture distributions. Stat. Methods Med. Res..

[31] Hibino S (2010). Approximation of ecg t wave by using gaussian mixtures and automatic measurement of qt interval. Trans. Jap. Soc. for Med Biol. Eng.

[32] Baum LE, Petrie T (1966). Statistical inference for probabilistic functions of finite state markov chains. Ann. Math. Stat..

[33] Werbos PJ (1990). Backpropagation through time: what it does and how to do it. Proc. IEEE.

[34] NIH Office of Extramural Research:Protecting Human Research Participants, howpublished = https://phrp.nihtraining.com/users/login.php. Accessed 01 May 2015.

[35] Inokuchi R (2013). The proportion of clinically relevant alarms decreases as patient clinical severity decreases in intensive care units: a pilot study. BMJ Open.

[36] Paine CW (2016). Systematic review of physiologic monitor alarm characteristics and pragmatic interventions to reduce alarm frequency. J. Hosp. Med..

[37] Ryu S (2002). Vagal activity increase and decrease, as a contributing factor in maintaining ventricular tachycardia. St. Marianna Med. J..

[38] Barra, O.A. & Moretti, L. The” life potential”: a new complex algorithm to assess” heart rate variability” from holter records for cognitive and diagnostic aims. preliminary experimental results showing its dependence on age, gender and health conditions. arXiv preprint arXiv:1310.7230 (2013).

[39] Brennan M, Palaniswami M, Kamen P (2001). Do existing measures of poincare plot geometry reflect nonlinear features of heart rate variability?. IEEE Trans. Biomed. Eng..

[40] Tarvainen MP, Niskanen JA, Lipponen JA, Ranta-aho PO, Karjalainen PO (2008). Kubios hrv—a software for advanced heart rate variability analysis. IFMBE Proc..

