# Supplementary material for: Recurrent probabilistic neural network-based short-term prediction for acute hypotension and ventricular fibrillation
Source: Sci Rep. 2020 Jul 20;10:11970. doi: 10.1038/s41598-020-68627-6 (PMC7371879; doi:10.1038/s41598-020-68627-6)
Supplement: Supplementary file 1 — Supplementary information. [file 41598_2020_68627_MOESM1_ESM.pdf]

Recurrent Probabilistic Neural Network-based Short-term Prediction for Acute Hypotension and Ventricular Fibrillation

Toshio Tsuji[1*], Tomonori Nobukawa[2], Akihisa Mito[2], Harutoyo Hirano[3], Zu Soh[1], Ryota Inokuchi[4], Etsunori Fujita[5], Yumi Ogura[5], Shigehiko Kaneko[6], Ryuji Nakamura[7], Noboru Saeki[7], Masashi Kawamoto[7], and Masao Yoshizumi[7]

[1]Faculty of Engineering, Hiroshima University, 1-4-1 Kagamiyama, Higashi-Hiroshima, Hiroshima 739-8527, Japan
[2]Graduate School of Engineering, Hiroshima University, 1-4-1 Kagamiyama, Higashi-Hiroshima, Hiroshima 739-8527, Japan
[3]Academic Institute, College of Engineering, Shizuoka University, 3-5-1, Johoku, Naka-ku, Hamamatsu, Shizuoka, Japan, 432-8561
[4]Department of Emergency and Critical Care Medicine, JR General Hospital, 2-1-3 Yoyogi, Shibuya-ku, Tokyo, 151-8528, Japan
[5]Delta Kogyo Co. Ltd., 1-14 Shinchi, Fuchu-Cho, Aki-Gun, Hiroshima, 735-8501, Japan
[6]Department of Mechanical Engineering, The University of Tokyo, 7-3-1 Hongo, Bunkyo-Ku, Tokyo, 113-8656, Japan
[7]Graduate School of Biomedical & Health Sciences, Hiroshima University, 1-2-3 Kasumi, Minami-Ku, Hiroshima, Hiroshima 734-8553, Japan
[*]tsuji@bsys.hiroshima-u.ac.jp

ABSTRACT

In this paper, we propose a novel method for predicting acute clinical deterioration triggered by hypotension, ventricular fibrillation, and an undiagnosed multiple disease condition using biological signals, such as heart rate, RR interval, and blood pressure. Efforts trying to predict such acute clinical deterioration events have received much attention from researchers lately, but most of them are targeted to a single symptom. The distinctive feature of the proposed method is that the occurrence of the event is manifested as a probability by applying a recurrent probabilistic neural network, which is embedded with a hidden Markov model and a Gaussian mixture model. Additionally, its machine learning scheme allows it to learn from the sample data and apply it to a wide range of symptoms. The performance of the proposed method was tested using a dataset provided by Physionet and the University of Tokyo Hospital. The results show that the proposed method has a prediction accuracy of 92.5% for patients with acute hypotension and can predict the occurrence of ventricular fibrillation 5 min before it occurs with an accuracy of 82.5%. In addition, a multiple disease condition can be predicted 7 min before they occur, with an accuracy of over 90%.

Supplementary Information

S1: Structure of recurrent log-linearised Gaussian mixture network

Supplementary Figure 1 shows the structure of the proposed recurrent log-linearised Gaussian mixture network (R-LLGMN). The R-LLGMN can learn the weight coefficients, which are the parameters employed for the prediction of acute clinical deterioration. The input vector $x(t)(t = 1,2,...,T_d; T_d$ is the time-series length of the input signal) is nonlinearly transformed according to the following equation for a new input vector $X(t)$ to be obtained.

$$X(t) = [1, x(t)^T, x_1(t)^2, x_1(t)x_2(t), \ldots, x_1(t)x_L(t), x_2(t)^2, x_2(t)x_3(t), \ldots, x_2(t)x_L(t), ..., x_L(t)^2]^T \quad (1)$$

where L is the dimension of the measured time-series signal. The first layer is composed of $H = 1 + L(L+3)/2$ units, and the identity function is used for the activation of each unit. $X_h(t)(h = 1,2,...,H)$ and ${}^{(1)}O_h(t)$ denote the input and output, respectively.

The second layer consists of $\{c,k,k',m\}(c = 1,...,C; k,k' = 1,...,K_c; m = 1,...,M_{c,k})$ units receiving the output of the first layer weighted by the coefficient $\omega^c_{k',k,m,h}$. $c = 1,2,...,C$, K_c and $M_{c,k}$ are the number of learning classes, number of states, and

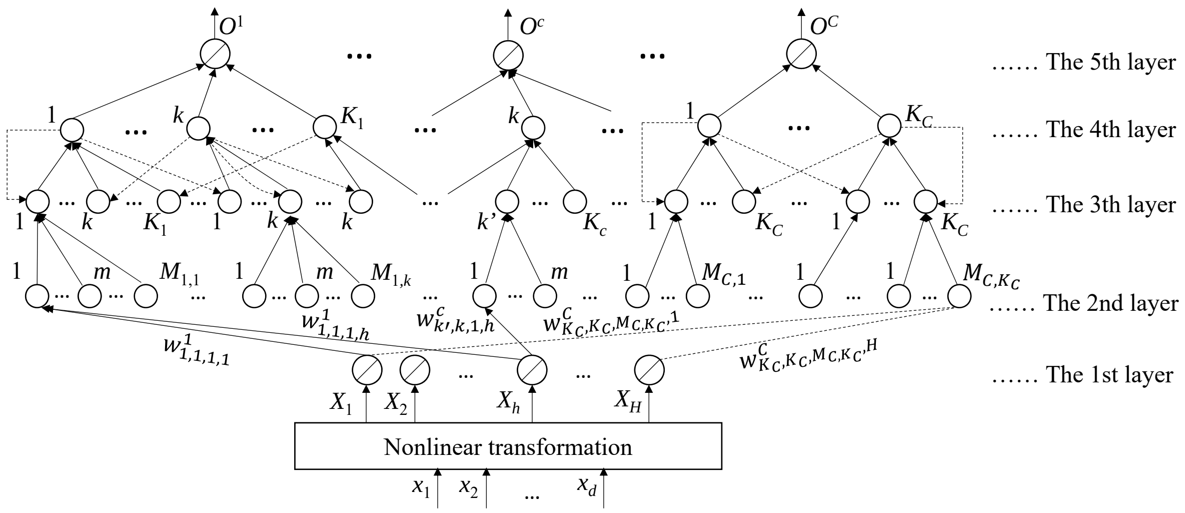

Supplementary Figure 1. Structure of R-LLGMN

number of components, respectively. The input ${}^{(2)}I^{c}_{k',k,m}(t)$ and ${}^{(2)}O^{c}_{k',k,m}(t)$ are defined as

$$ {}^{(2)}I^{c}_{k',k,m}(t) = \sum_{h=1}^{H} {}^{(1)}O_{h}(t)\omega^{c}_{k',k,m,h} \tag{2} $$

$$ {}^{(2)}O^{c}_{k',k,m}(t) = \exp({}^{(2)}I^{c}_{k',k,m}(t)). \tag{3} $$

The output of the second layer $\{c,k,k',m\}(m = 1,...,M_{c,k})$ is added up and input to the third layer $\{c,k,k'\}$. In addition, the output of the fourth layer is fed back to the third layer. These processes are expressed as follows:

$$ {}^{(3)}I^{c}_{k',k}(t) = \sum_{m=1}^{M_{c,k}} {}^{(2)}O^{c}_{k',k,m}(t) \tag{4} $$

$$ {}^{(3)}O^{c}_{k',k}(t) = {}^{(4)}O^{c}_{k'}(t-1)\,{}^{(3)}I^{c}_{k',k}(t) \tag{5} $$

where ${}^{(4)}O^{c}_{k'}(0) = 1.0$ for the initial iteration of learning. The activation functions in the fourth layer are described as follows:

$$ {}^{(4)}I^{c}_{k}(t) = \sum_{k'=1}^{K_c} {}^{(3)}O^{c}_{k',k}(t) \tag{6} $$

$$ {}^{(4)}O^{c}_{k}(t) = \frac{{}^{(4)}I^{c}_{k}(t)}{\sum_{c'=1}^{C}\sum_{k'=1}^{K_{c'}} {}^{(4)}I^{c'}_{k'}(t)}. \tag{7} $$

Finally, the unit c in the fifth layer accumulates the outputs of K_c units $\{c,k\}(k = 1,...,K_c)$ from the fourth layer. The relationship in the fifth layer is defined as

$$ {}^{(5)}I^{c}(t) = \sum_{k=1}^{K_c} {}^{(4)}O^{c}_{k}(t) \tag{8} $$

$$ {}^{(5)}O^{c}(t) = {}^{(5)}I^{c}(t). \tag{9} $$

As the output ${}^{(5)}O^c(t)$ corresponds to the calculation of the posterior probability via Bayes' theorem, the posterior probability, which employs the feature vectors x consisting of c, can be calculated internally by the R-LLGMN. On the basis of the supervised learning paradigm, the weight factors between the first and second layers are adjusted such that the posterior probability of each class can be calculated. In this study, two classes have been defined, namely, the normal (c=1) and event (c=2) classes. An event can be predicted P minutes before it actually occurs. Let us represent a pair of learning data for the R-LLGMN, consisting of the input vector of $\boldsymbol{x}_c^{(n)}$=[$\boldsymbol{x}_c^{(n)}(1)$, $\boldsymbol{x}_c^{(n)}(2)$, ..., $\boldsymbol{x}_c^{(n)}(t)$, ...,$\boldsymbol{x}_c^{(n)}(T_d)$] where $\boldsymbol{x}_c^{(n)}(t)$=$[x_{1,c}^{(n)}(t),...,x_{i,c}^{(n)}(t),...,x_{I,c}^{(n)}(t)]^T$ and the corresponding teacher vector $Y^{(n)} = [Y_1^{(n)},...,Y_c^{(n)},...,Y_C^{(n)}]^T$. Here, the element ${x_{i,c}}^{(n)}(t)$ is a biological signal measured at time t, and $Y_c^{(n)}$ is the posterior probability of class c. Each index represents the following: $n = 1,2,...,N$ is the dataset number, T_d is the total time step to output a posterior probability vector ${O_c}^{(n)}$ at the output layer of the R-LLGMN, class $c = 1$ represents the class for a normal condition, and $c = 2$ represents the class for acute clinical deterioration, such that $C = 2$. Given that N pairs of learning data were prepared for each class c, the total of N times C pairs of learning data were used to adjust the parameters. The evaluation function J is then defined as follows:

$$J = \sum_{n=1}^{N} J_n = -\sum_{n=1}^{N}\sum_{c=1}^{C} Y_c^{(n)} \log^{(5)} O^c(T)^{(n)}. \tag{10}$$

The learning process minimizes the above function, which implies a maximization of the likelihood. The weight modification $\Delta\omega^c_{k',k,m,h}$ for $\omega^c_{k',k,m,h}$ is defined as

$$\begin{aligned}
\Delta\omega^c_{k',k,m,h} &= -\eta \sum_{n=1}^{N} \frac{\partial J_n}{\partial \omega^c_{k',k,m,h}} \\
&= -\sum_{t=1}^{T-1}\sum_{c'=1}^{C}\sum_{k''=1}^{K_{c'}} \Delta^{c'}_{k''}(t) \\
&\times(\delta_{(c',k''),(c,k)} - {}^{(4)}O^{k'}_{k''}(T-t)) \\
&\times \frac{{}^{(4)}O^{c'}_{k''}(T-t)}{{}^{(4)}I^{c'}_{k''}(T-t)} {}^{(4)}O^{c}_{k'}(T-t-1) \\
&\times {}^{(2)}O^{c}_{k',k,m}(T-t)X_h(T-t)
\end{aligned} \tag{11}$$

in a collective learning scheme with a fixed $\eta > 0$ as the learning rate. $\delta_{(c',k''),(c,k)}$ is defined as

$$\delta_{(c',k''),(c,k)} = \begin{cases} 1 & (c' = c; k'' = k) \\ 0 & (otherwise) \end{cases} \tag{12}$$

where $\Delta^{c'}_{k''}(t)$ represents a partial differentiation with ${}^{(4)}O^{c'}_{k''}(T-t)$, ${}^{(5)}O^c(T)^{(n)}$, which signifies the output at time T for the input $x(t)^{(n)}$. As per the abovementioned learning process, the parameters of the Gaussian mixture model were determined.

Binary and categorical data are often provided in clinical practice; the ability to treat these data correctly is important. The binary data can be input to the R-LLGMN via centering and scaling with a mean of 0 and a standard deviation of 1. By means of such preprocessing, the Gaussian mixture distribution can approximate the binary data in the same manner as it approximates the continuous data. The category data can also be treated by extending the abovementioned preprocessing. Given data with E categories, one can configure an E-dimensional dummy variable $x_{\mathrm{d},1}, x_{\mathrm{d},2}, \cdots, x_{\mathrm{d},E}$ and express the presence and absence of a category by setting the corresponding dummy variable to 1 and 0, respectively. Because the R-LLGMN does not require the calculation of an inversed covariance matrix, the number of dummy variables can be equal to the category number, but it can also be set to $E-1$, considering the degrees of freedom. Incorporation of the discrete signals generated by the clinical apparatus may help to improve the prediction accuracies.

S2: Definition of sensitivity, specificity, and accuracy

Sensitivity is the proportion of true positives that are correctly predicted by the proposed method; specificity is the proportion of true negatives that are predicted correctly, and accuracy is the proportion of all subjects that are predicted correctly. In general, sensitivity, specificity, and accuracy are calculated as follows:

$$\text{Sensitivity [\%]} = \frac{\text{The number of true positives}}{\text{The number of true positives} + \text{The number of false negatives}} \times 100 \tag{13}$$

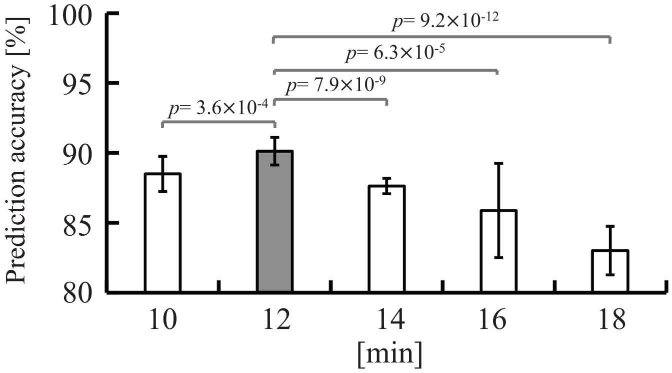

Supplementary Figure 2. Average prediction accuracy by each window period.

$$\text{Specificity [\%]} = \frac{\text{The number of true negatives}}{\text{The number of true negatives} + \text{The number of false positives}} \times 100 \tag{14}$$

$$\text{Accuracy [\%]} = \frac{\text{The number of true negatives} + \text{The number of true positives}}{\text{The number of all subjects}} \times 100. \tag{15}$$

In the study, we compared the proposed method with the previous methods on the basis of these performance parameters.

S3: Prediction window period selection

The dataset used here was Dataset 1, as shown in Table 1. This dataset consists of the data of 30 patients who developed acute hypotension and 30 patients who did not. The window period was altered by 2 min from 10 to 18 minutes, and the prediction accuracy of each window period was calculated via leave-one-subject-out cross-validation of 60 patients. A multiple comparison test using Bonferroni correction was then conducted to determine if there were significant differences among the average accuracies of the five window periods.

Supplementary Figure 2 shows the average prediction accuracies of the five window periods: 88.5±1.3%, 90.1±1.0%, 87.6±0.5%, 85.9±3.4%, and 83.0±1.7%. For a window period of 12 min, the average prediction accuracy was significantly higher than that of all other window periods. On the basis of the above result, a prediction window period of 12 min was considered in the section titled "Prediction of acute hypotension occurrence" in the main paper.

S4: Prediction of acute hypotension using the other test set by Physionet Challenge 2009

In addition to conducting the experiments detailed in the paper, we conducted another experiment involving the prediction of acute hypotension using the other test set provided by Physionet Challenge 2009. The test set consists of 10 patients (five patients with acute hypotension and five normal patients) for verification. The training set and R-LLGMN hyperparameters of the proposed method are the same as those used for the R-LLGMN hyperparameter selection. A sampling time of 1 min was considered, and the input signals were of heart rate, systolic blood pressure, diastolic blood pressure, and mean blood pressure.

Supplementary Table 1 compares the prediction results of the proposed method and those of Henriques *et al.*'s method, which achieved the highest accuracy among all the methods published by Physionet Challenge, i.e., the same method as that used for prediction of acute clinical deterioration triggered by acute hypotension in the main manuscript. The table shows that the proposed method as well as Henriques *et al.*'s method made correct predictions for all 10 patients. The number of true positives, true negatives, false positives, and false negatives predicted via the proposed method were the same as those predicted by Henriques *et al.*'s method. Therefore, the sensitivity, specificity, and accuracy values were also the same.

Supplementary Table 1. Comparison between proposed method and Henriques *et al.*'s method using the other test set.

	True positives	False positives	True negatives	False negatives	Sensitivity [%]	Specificity [%]	Accuracy [%]
The proposed method	5	0	5	0	100.0	100.0	100.0
Henriques *et al.* method	5	0	5	0	100.0	100.0	100.0
